# Adverse Neurological Effects of Short-Term Sleep Deprivation in Aging Mice Are Prevented by SS31 Peptide

**DOI:** 10.3390/clockssleep2030024

**Published:** 2020-08-06

**Authors:** Jinzi Wu, Yan Dou, Warren C. Ladiges

**Affiliations:** Department of Comparative Medicine, School of Medicine, University of Washington, Seattle, WA 98195, USA; jinziwu90@163.com (J.W.); ydou@uw.edu (Y.D.)

**Keywords:** aging, sleep deprivation, learning impairment, SS31, aged mouse model

## Abstract

Sleep deprivation is a potent stress factor that disrupts regulatory pathways in the brain resulting in cognitive dysfunction and increased risk of neurodegenerative disease with increasing age. Prevention of the adverse effects of sleep deprivation could be beneficial in older individuals by restoring healthy brain function. We report here on the ability of SS31, a mitochondrial specific peptide, to attenuate the negative neurological effects of short-term sleep deprivation in aging mice. C57BL/6 female mice, 20 months old, were subcutaneously injected with SS31 (3 mg/kg) or saline daily for four days. Sleep deprivation was 4 h daily for the last two days of SS31 treatment. Mice were immediately tested for learning ability followed by collection of brain and other tissues. In sleep deprived mice treated with SS31, learning impairment was prevented, brain mitochondrial ATP levels and synaptic plasticity regulatory proteins were restored, and reactive oxygen species (ROS) and inflammatory cytokines levels were decreased in the hippocampus. This observation suggests possible therapeutic benefits of SS31 for alleviating adverse neurological effects of short-term sleep loss.

## 1. Introduction

Studies have shown that sleep plays a vital role in brain plasticity and memory [1,2] and ensures efficacy of electrical firing within the neuronal synapse [3,4]. Disruption of normal sleep patterns triggers inflammatory pathways and interferes with synaptic transmission in the hippocampus, a specific area of the brain crucial for encoding and memory storage [5,6]. Sleep deprivation has been shown to induce mitochondrial dysfunction that leads to increased reactive oxygen species (ROS) and decreased ATP production [7]. The neurological damage induced by sleep deprivation is especially prevalent in the elderly due to increased incidence of sleep disorders and exaggerated neuroinflammatory responses [8,9,10,11,12]. As a result, finding a rational treatment for sleep deprivation could be highly beneficial by decreasing the risk for neurodegenerative conditions such as Alzheimer’s disease.

SS31 (D-Arg-dimethylTyr-Lys-Phe-NH2), a synthetic mitochondrial-specific peptide, has been shown to improve synaptic and cognitive impairments in mice and rats exposed to lipopolysaccharide and isoflurane [13,14,15]. The mechanism was mainly through reducing neuroinflammation and oxidative stress in the hippocampus, protecting the hippocampal neuron mitochondria, and enhancing synaptic plasticity and its related signaling pathways. In another study, aged mice treated with SS31 displayed significantly improved neurovascular coupling responses and cognitive functions, including spatial working memory and motor skill learning [16]. The effect of SS31 on learning impairment associated with short-term sleep deprivation in aging mice has not yet been investigated. We show in this report that SS31 prevented learning impairment in old mice after short-term sleep deprivation, and provide data suggesting the protective effects are mediated by preserving mitochondrial integrity and synaptic function.

## 2. Results

### 2.1. Sleep-Deprived Learning Impairment Was Prevented in Aging Mice Treated with SS31

**Figure 1:**
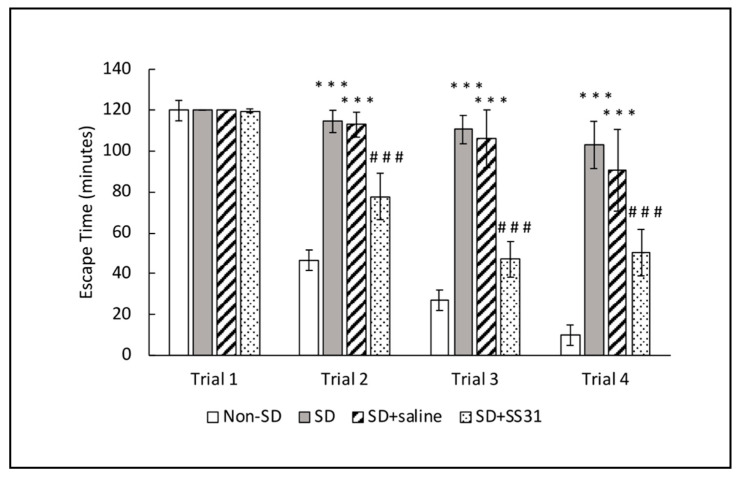
Learning impairment in aging mice induced by sleep deprivation was prevented with SS31. Mice were given four trials to escape the box maze for a maximum of 2 min each, and their escape times were recorded. The data represent the mean ± SEM (*n* = 10 per group). *** *p* < 0.001, statistically significant difference between SD group/SD + Saline group and Non-SD group. ### *p* < 0.001, statistically significant difference between SD + SS31 group and SD group/SD + Saline group.

In order to determine if SS31 could prevent learning deficits induced by short-term sleep deprivation (SD), 20-month-old mice were treated with SS31 (3 mg/kg) by subcutaneous injection once daily for four days. On the third and fourth days of treatment, mice were sleep deprived for 4 h each day. Immediately following the last sleep deprivation session, a spatial navigation task, designated as the box maze [17,18] was used to measure learning impairment, with escape time as the readout. Non-SD mice showed robust learning ability as indicated by the decreased escape times starting in trial 2 compared to SD mice (Figure 1). When SD mice were treated with SS31, learning impairment was significantly alleviated and more closely resembled learning ability in non-SD mice. These results suggest that SS31 can prevent learning impairment induced by short-term sleep deprivation in aging mice.

### 2.2. ATP Increased and ROS Decreased in Mitochondria from Brain and Liver of Sleep-Deprived Mice Treated with SS31

**Figure 2:**
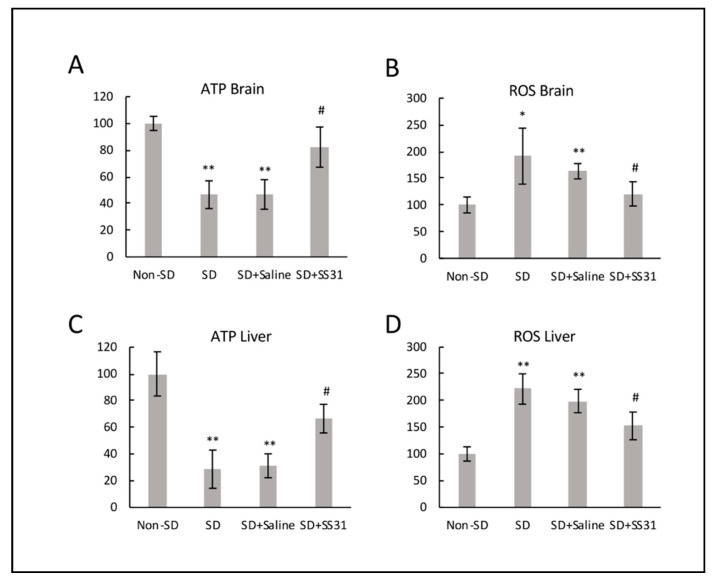
Mitochondria from brain and liver of sleep-deprived mice treated with SS31 had restored ATP and decreased reactive oxygen species (ROS). ROS and ATP levels were measured using isolated mitochondria from brain and liver. The *y*-axis shows percentages, with the non-SD group being 100 percent. (**A**) ATP level in brain tissue, (**B**) ROS level in brain tissue, (**C**) ATP level in the liver tissue, and (**D**) ROS level in liver tissue. All data represent the mean ± SEM of three independent triplicate experiments. ** *p* < 0.01, * *p* < 0.05, statistically significant difference between SD group/SD + Saline group and Non-SD group. # *p* < 0.05, statistically significant difference between SD + SS31 group and SD group/SD + Saline group.

Sleep-deprived (SD) mice had a significant decrease in ATP synthesis compared to non-SD mice in the brain (Figure 2A), suggesting disruption of mitochondrial function and inefficient energy production. On the other hand, ATP levels were restored in SD mice treated with SS31 to levels similar to non-SD mice. In order to determine if there was an increase in ROS that might be related, we measured ROS production in the brain and showed that SD mice had significantly higher levels of mitochondrial ROS in the brain compared to non-SD mice (Figure 2B), suggesting a correlation with disruption of ATP production. SD mice treated with SS31 showed a significant decrease in ROS, with levels similar to non-SD mice, further indicating the disruptive sensitivity of mitochondria to sleep deprivation. We also interrogated the liver for the above molecules in order to determine if mitochondrial integrity affected by SD in nonneuronal systemic tissues might be restored by SS31, thus raising the possibility of an indirect impact neuronal function. Results were similar to that seen in the brain, with SD causing lower ATP levels and higher levels of ROS (Figure 2C,D), both of which were rescued by treatment with SS31.

### 2.3. SS31 Restores Hippocampal Regulatory Proteins for Synaptic Plasticity, and Decreases Inflammatory Cytokines in Sleep-Deprived Mice

**Figure 3:**
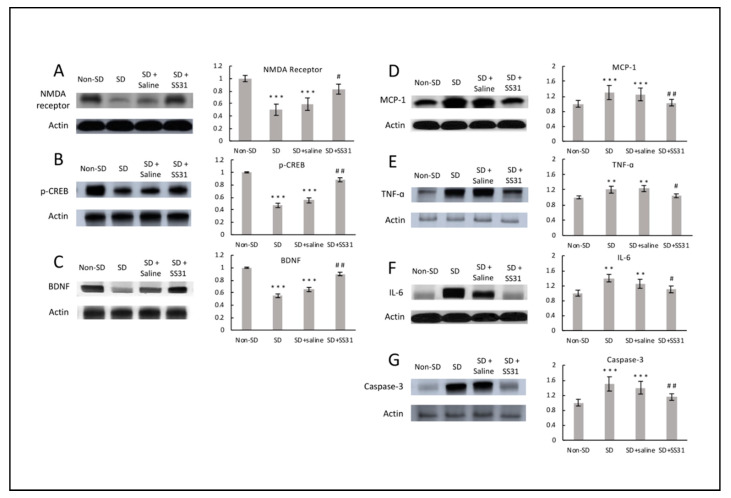
Hippocampal regulatory proteins for synaptic plasticity were restored, and inflammatory cytokines decreased in sleep-deprived mice treated with SS31. Western blotting was performed on hippocampus using specific antibodies for the detection of (**A**) NMDA receptor, (**B**) p-CREB, (**C**) BDNF, (**D**) MCP-1, (**E**) TNF-α, (**F**) IL-6, and (**G**) caspase-3, with Actin used as loading control. The densitometry values for the proteins were normalized to those of Actin, with non-SD group being 1. All data represent the mean ± SEM (*n* = 5 per group). ** *p* < 0.01, *** *p* < 0.001, statistically significant difference between SD group/SD + Saline group and Non-SD group. # *p* < 0.05, ## *p* < 0.01, statistically significant difference between SD + SS31 group and SD group/SD + Saline group.

To investigate molecular players associated with SS31-attenuated learning impairment induced by sleep deprivation in aging mice, we tested expression levels of three known regulators of synaptic plasticity. *N*-methyl-D-aspartate (NMDA) receptor is a glutamate receptor that plays a vital role in regulating synaptic plasticity and subsequent function in the hippocampus [4,19]. CREB (cAMP-response element binding), a transcription factor downstream of the cAMP/PKA signaling pathway, and brain-derived neurotrophic factor (BDNF) are other regulators of synaptic plasticity, playing vital roles for learning and memory [3,20,21]. Compared to non-SD mice, SD mice had significantly lower levels of NMDA receptor, p-CREB, and BDNF (Figure 3A–C), indicating the negative effects of short-term sleep deprivation on synaptic plasticity-related regulation might be the result of high sensitivity to ROS-mediated inflammation in the brain. Treatment with SS31 greatly attenuated the decrease in plasticity regulator protein expression induced by sleep deprivation, suggesting synaptic plasticity can be modulated by SS31.

Since neuroinflammation caused by sleep deprivation is associated with synaptic impairment, especially in the hippocampus [6,22], we examined effects of SS31 on hippocampal inflammation. Levels of three inflammatory cytokines, MCP-1, TNF-α, and IL-6, were measured. Compared to non-SD mice, SD mice had significantly higher levels of MCP-1, TNF-α, and IL-6 expression (Figure 3D–F), which might have been due to increased neuronal ROS. Cytokines such as TNF-α and IL-6 have been shown to induce changes in hippocampal dependent learning and memory tasks [23]. Increased hippocampal inflammation in relation to sleep deprivation has been well studied, and the effect of neuroinflammation on cognitive function can be detrimental [6,13,22]. SD mice treated with SS31 had a significant decrease in hippocampal inflammation, to a level similar to non-SD mice, suggesting that loss of mitochondrial integrity appears to be related to the increased inflammation caused by short-term sleep deprivation in aging mice.

Because an increase in inflammation can trigger an apoptotic response, we tested for caspase-3, an enzyme known to be involved in the apoptotic pathway [24]. Mice that were sleep deprived had significantly elevated levels of caspase-3 expression in the hippocampus compared to non-SD mice (Figure 3G), indicating activation of the apoptotic neuronal cell death pathway in response to short-term sleep deprivation. Caspase-3 was greatly reduced in SD mice treated with SS31, suggesting a relationship between sleep deprivation-induced mitochondrial dysfunction, neuroinflammation, and elimination of compromised neuronal cells.

## 3. Discussion

Aging C57BL/6 mice, treated with the mitochondrial-targeted peptide SS31 daily for 4 days and sleep deprived on the third and fourth days of treatment, do not show the learning impairment seen in age-matched sleep deprived control mice but have nearly normal learning ability similar to non-sleep deprived mice. Sleep deprived mice treated with SS31 showed suppression of mitochondrial ROS and enhanced ATP production in the brain and liver. These mice also exhibited a near reversal of damaged synaptic plasticity regulation and decreased inflammation in the hippocampus caused by short-term sleep deprivation.

The increased ROS in neuronal cells of sleep deprived mice may have triggered the increased inflammatory cytokine expression in the hippocampus. Cytokines such as TNF-α and IL-6 have been shown to induce changes in hippocampal dependent learning and memory tasks [23]. Increased hippocampal inflammation in relation to sleep deprivation has been well studied, and the effect of neuroinflammation on cognitive function can be detrimental [6,13,22]. Levels of TNF-α, IL-6 and the inflammatory mediator MCP-1 were significantly increased in the hippocampus of sleep deprived mice compared to non-sleep deprived mice, providing an inflammatory environment for learning impairment. Since SS31 significantly suppressed the sleep deprivation-induced inflammation in the hippocampus, mitochondrial dysfunction appears to be related to the increased inflammation caused by short-term sleep deprivation in aging mice.

The high mitochondrial presence in synapses would suggest that synaptic function would be especially sensitive to ROS-mediated inflammation in the brain. Our results suggest that this could explain the decreased expression of vital synaptic plasticity regulators such as NMDA receptor, p-CREB, and BDNF, contributing to the learning impairment observed in sleep deprived mice. Concurrently, increased presence of ROS could be overwhelming the neuronal antioxidant capacity, resulting in cellular damage [25] and activation of the apoptotic pathway [26] as shown by increased expression of caspase-3 in sleep deprived mice. Therefore, the increased expression of inflammatory cytokines induced by sleep deprivation might be compromising neuronal function by damaging vital cellular components.

In addition, sleep deprivation has also been shown to increase inflammation and oxidative stress in multiple organs, including the liver which plays a central role in regulating metabolism [27]. Our observation of increased ROS and decreased ATP levels in liver mitochondria support the negative effect of sleep deprivation on hepatic metabolism, which could indirectly affect neurological function in the hippocampus [28,29]. Since treatment with SS31 greatly attenuated sleep deprived learning impairment, it is possible that non-neuronal systemic effects of SS31 may have contributed to the improved cognitive ability.

Although we do not have data to confirm a mechanistic sequence of events, it is possible that the sleep deprivation-induced increase in ROS related to mitochondrial dysfunction started a molecular cascade of neuronal dysfunction leading to learning impairment. SS31 is a free radical scavenger with an ability to interact with the inner mitochondrial membrane to modulate electron flux, increase ATP generation, and decrease ROS production [30,31]. Since treatment with SS31 suppressed the detrimental effect of sleep deprivation in aging mice, it is logical to suggest a cause and effect relationship between sleep deprivation-induced mitochondrial dysfunction, oxidative stress, and learning impairment.

In summary, our studies have linked sleep deprivation with SS31 and aging. The data show that SS31 is associated with the prevention of sleep-deprived adverse changes in cognitive function, mitochondrial ROS production and energy output, regulation of synaptic plasticity, and inflammatory pathways in aging mice. The results provide the rationale to further investigate SS31 as a novel clinical therapeutic for age-related short-term sleep deprivation. The study sheds light on the possible therapeutic benefits of SS31 for cognitive decline with increasing age and short-term sleep loss.

## 4. Methods

### 4.1. Animals and Treatment Schedule

C57BL/6 female mice, 20 months of age, were obtained from the Aging Rodent Colony of the National Institute on Aging managed by Charles River Inc. Animals were maintained in a specific pathogen free housing facility at the University of Washington with standard rodent chow ad lib, reverse osmosis water via an automatic watering system, and room temperature maintained at 70–72 °F. Mice were allowed to acclimate to the housing conditions for at least two weeks before entering into any experiments. All animal experiments were approved by the University of Washington Institutional Animal Care and Use Committee.

Mice were treated with SS31 (Stealth BioTherapeutics, Newton, MA, USA) by subcutaneous injection at a dose of 3 mg/kg once daily for four days. On the third and fourth days of treatment, mice were sleep deprived for 4 h each day. Immediately following the last sleep deprivation session, a spatial navigation task was used to measure learning impairment.

### 4.2. Sleep Deprivation Procedure

The sleep deprivation procedure was carried out as previously described [17]. Based on a 12:12 dark/light cycle, the animals were sleep deprived starting 4 h after the lights came on for a total of 4 h daily for 2 days. Sleep deprivation was achieved through continual low stress sleep disturbances including cage tapping and gentle stroking of the back with a small brush. The Box maze assay was conducted immediately following the last sleep deprivation session.

### 4.3. Spatial Navigation Box Maze

The box maze spatial navigation task was conducted as previously described [18] to determine learning impairment caused by sleep deprivation. Briefly, the box maze consists of a rectangular clear hard-plastic box (26.5 cm width, 30.5 cm length and 29.2 cm height). Each side of the box has two holes and each hole has a distinctive decoration placed above it. The holes were placed and centered 3 cm from the bottom of the cage. During the procedure, 7 of the holes were blocked with one escape hole open to a tube leading to an escape cage. Testing consisted of four 120 s trials. A trial was scored as completed when all four paws were inside the escape hole. The time (latency) to complete the trial was then recorded. If the mouse was unable to find the escape hole it was shown the escape hole and given a latency time of 120 s. Between trials, odor markers were removed from the maze with 70% ethanol.

### 4.4. Western Blot

Hippocampus was isolated from the mouse brain within 2 min of euthanasia, and snap frozen at −80 °C to be used for Western blots. Protein extraction was carried out using T-PER^TM^ Tissue Protein Extraction Reagent (ThermoFisher Scientific, Waltham, MA, USA, #78510). After the transferring, blots were blocked in 5% *w/v* BSA, 1× TBS, 0.1% Tween at room temperature for 1 h; afterwards the blots were incubated with primary antibody in 5% *w/v* BSA, 1× TBS, 0.1% Tween at 4 °C overnight with gentle shaking. The blots were developed using enhanced chemiluminescence (ECL) immunochemical detection kit (Bio-Rad, Richmond, CA, USA) and densitometric analysis was conducted to quantify the Western blot immunoreactivity with a scanner and ImageQuant software (Amersham Biosciences, Waltham, MA, USA). The primary antibodies used were N-methyl-D-aspartate (NMDA) receptor (1:1000, Cell Signaling Technology, Danvers, MA, USA, #4207), p-CREB (1:1000, Cell Signaling Technology, #9198), caspase-3 (1:1000, Cell Signaling Technology, #9662S), BDNF (1:1000, Abcam, Cambridge, UK, ab203573), TNF-α (1:500, Novus Biologicals, Littleton, CO, USA, NBP1-19532), IL-6 (1:1000, Novus Biologicals, NB600-1131), CCL2/MCP1 (1:1000, Novus Biologicals, NBP-07035) and actin (1:1000, Cell Signaling Technology, 4967). The secondary antibody used was goat anti-rabbit IgG-HRP (1:4000, Santa Cruz Biotechnology, Santa Cruz, CA, USA).

### 4.5. Isolation of Mitochondria

Brain. Mitochondria isolation from the whole brain was carried out using Percoll gradient centrifugation as previously reported [32] with slight modifications [33,34]. Brains were removed rapidly and homogenized in 15 mL of ice-cold mitochondrial isolation buffer containing 0.32 M sucrose, 1 mM EDTA and 10 mM Tris-HCl, pH 7.1. The homogenate was centrifuged at 1330× *g* for 10 min and the supernatant was saved. The pellet was resuspended in half volume (7.5 mL) of the original isolation buffer and centrifuged again under the same conditions. The two supernatants were combined and centrifuged further at 21,200× *g* for 10 min. The resulting pellet was resuspended in 12% Percoll solution (Fisher Scientific, 45-001-74) prepared in mitochondrial isolation buffer followed by centrifugation at 6900× *g* for 10 min. The obtained soft pellet was resuspended in 10 mL of the mitochondrial isolation buffer and centrifuged again at 6900× *g* for 10 min. All of the mitochondrial pellets obtained after centrifugation were either used immediately or frozen at −80 °C until analysis. The protein concentration was determined by the Pierce BCA assay kit (ThermoFisher Scientific, #23227).

Liver. Mitochondria from the liver were isolated according to a previous described method [35] with slight modifications. Liver tissues were homogenized (1 g/10 mL isolation buffer) in mitochondrial isolation buffer containing 15 mM MOPS (pH 7.2), 70 mM sucrose, 230 mM mannitol, and 1 mM K^+^-EDTA. The homogenates were centrifuged at 8000× *g* for 10 min at 4 °C. The resulting supernatant was further centrifuged at 8000× *g* for 10 min at 4 °C. The resulting pellet containing crude mitochondria was washed with 10 mL of the isolation buffer followed by centrifugation under the same conditions. The obtained mitochondrial pellet was either used immediately or frozen at −80 °C until further use. The protein concentration was determined by the Pierce BCA assay kit (ThermoFisher Scientific, #23227).

### 4.6. ROS Assay

The ROS assay was carried out using mitochondria isolated from liver and brain with the reactive oxygen species assay kit (MyBioSource, San Diego, CA, USA, MBS2540517) according to the manufacturer’s protocol manual. DCFH-DA (2,7-Dichlorofluorescein diacetate) is a fluorescent probe without fluorescence that can freely cross the cell membrane and can be hydrolyzed by intracellular esterase to form DCFH (dichlorofluorescin). In the presence of ROS, DCFH is oxidized to DCF (dichlorofluorescein) which is a strong green fluorescent substance that cannot penetrate the cell membrane. In short, 20 uM of DCFH-DA working solution was added to the mitochondrial pellet and incubated for 1 h at 37 °C. The mitochondrial suspension was then centrifuged, and the pellets were washed two times with the working reagent from the kit, then resuspended in the working reagent with equal volume and equal protein concentration on a microplate for fluorescence detection. Fluorescence at 500 nm excitation and 525 nm emission was then read on a fluorescence microplate reader (Synergy H1, BioTek, Winooski, VT, USA).

### 4.7. ATP Assay

The ATP assay was carried out using mitochondria isolated from liver and brain with the ATP Colorimetric/Fluorometric Assay Kit (BioVision, Milpitas, CA, USA, K354-100) according to the manufacturer’s protocol manual. In short, 50 uL of ATP assay reaction mix was added to 50 uL of mitochondrial suspension on a microplate and incubated at room temperature for 30 min. Fluorescence at 535 nm excitation and 587 nm emission was then read on a fluorescence microplate reader (Synergy H1, BioTek, Winooski, VT, USA).

### 4.8. Statistical Analysis

All the data are presented as mean ± SEM. Statistical comparisons were performed with the independent t-test. The criterion for statistical significance was considered to be *p* < 0.05. When comparing between SD group/SD + Saline group and Non-SD group, two independent *t*-tests were performed total between SD group and Non-SD group, and between SD + Saline group and Non-SD group.
